# Editorial Comment on “Successful Closure of Persistent Urinary Fistula After High‐Grade Renal Trauma Using N‐Butyl‐2‐Cyanoacrylate: A Case Report”

**DOI:** 10.1002/iju5.70258

**Published:** 2026-09-02

**Authors:** Ryoichi Kitamura

**Affiliations:** ^1^ Tajima Emergency and Critical Care Medical Center Toyooka Public Hospital Toyooka Hyogo Japan

**Keywords:** cyanoacrylates, embolization, kidney injuries, nephrectomy, therapeutic, urinary fistula

Yamauchi et al. report preservation of the injured kidney in an 18‐year‐old patient with persistent urinary leakage after Grade IV renal trauma. Despite ureteral stenting and percutaneous drainage, the fistula persisted for more than 60 days. Instead of proceeding directly to reconstruction or nephrectomy, the authors assessed the fistulous anatomy and achieved closure using coils and N‐butyl‐2‐cyanoacrylate (NBCA). Their persistence and ingenuity are commendable [1].

Kidney preservation is central in renal trauma care. Hemodynamic stability largely determines initial management, and many stable patients with high‐grade injuries can be managed non‐operatively [2]. Most urinary extravasation resolves spontaneously. For persistent leakage requiring intervention, ureteral stenting is commonly used; percutaneous drainage may be added for a symptomatic or infected urinoma [2].

Several salvage strategies have been described. When an injured renal segment remains perfused but has lost normal drainage into the collecting system, selective arterial embolization can stop urine production from that segment, although functioning parenchyma is sacrificed [3]. Focal cryoablation has also been reported in postoperative cases [4]. Other approaches directly target the leak. When a fistulous tract is identified, coils may occlude the tract and serve as a scaffold for NBCA. When no distinct tract is identified, percutaneous NBCA sealing of the urinoma cavity has been reported [5].

The feasibility of direct fistula embolization depends largely on the anatomy of the leak. It may be considered when the tract is clearly delineated and safely accessible and when migration of embolic material into the collecting system can be prevented. If these conditions are not met, direct embolization may be technically difficult or unsafe. NBCA migration into the collecting system may result in cast formation and urinary tract obstruction; protective techniques are therefore important.

Selective fistula embolization should not replace established initial management. Rather, it may be considered a salvage option in selected patients with persistent leakage despite ureteral stenting and, when indicated, drainage, provided that preservation of the injured kidney remains feasible. In this patient, successful closure without nephrectomy highlights the value of pursuing kidney preservation before more invasive surgery when safely feasible.

## Conflicts of Interest

The author declares no conflicts of interest.

## Data Availability

The author has nothing to report.
